# Ascorbic acid promotes cardiomyogenesis through SMAD1 signaling in differentiating mouse embryonic stem cells

**DOI:** 10.1371/journal.pone.0188569

**Published:** 2017-12-12

**Authors:** Maria Grazia Perino, Satoshi Yamanaka, Daniel R. Riordon, Yelena Tarasova, Kenneth R. Boheler

**Affiliations:** 1 Laboratory of Cardiovascular Science, National Institute on Aging, National Institutes of Health, Baltimore, Maryland, United States of America; 2 Stem Cell and Regenerative Medicine Consortium, School of Biomedical Sciences, LKS Faculty of Medicine, University of Hong Kong, Hong Kong, SAR China; 3 Division of Cardiology, Johns Hopkins Medical Institute, Baltimore, Maryland, United States of America; University of Texas at Austin Dell Medical School, UNITED STATES

## Abstract

Numerous groups have documented that Ascorbic Acid (AA) promotes cardiomyocyte differentiation from both mouse and human ESCs and iPSCs. AA is now considered indispensable for the routine production of hPSC-cardiomyocytes (CMs) using defined media; however, the mechanisms involved with the inductive process are poorly understood. Using a genetically modified mouse embryonic stem cell (mESC) line containing a dsRED transgene driven by the cardiac-restricted portion of the *ncx1* promoter, we show that AA promoted differentiation of mESCs to CMs in a dose- and time-dependent manner. Treatment of mPSCs with AA did not modulate total SMAD content; however, the phosphorylated/active forms of SMAD2 and SMAD1/5/8 were significantly elevated. Co-administration of the SMAD2/3 activator Activin A with AA had no significant effect, but the addition of the nodal co-receptor TDGF1 (Cripto) antagonized AA’s cardiomyogenic-promoting ability. AA could also reverse some of the inhibitory effects on cardiomyogenesis of ALK/SMAD2 inhibition by SB431542, a TGFβ pathway inhibitor. Treatment with BMP2 and AA strongly amplified the positive cardiomyogenic effects of SMAD1/5/8 in a dose-dependent manner. AA could not, however, rescue dorsomorphin-mediated inhibition of ALK/SMAD1 activity. Using an inducible model system, we found that SMAD1, but not SMAD2, was essential for AA to promote the formation of TNNT2^+^-CMs. These data firmly demonstrate that BMP receptor-activated SMADs, preferential to TGFβ receptor-activated SMADs, are necessary to promote AA stimulated cardiomyogenesis. AA-enhanced cardiomyogenesis thus relies on the ability of AA to modulate the ratio of SMAD signaling among the TGFβ-superfamily receptor signaling pathways.

## Introduction

Human pluripotent stem cells (hPSCs) hold great promise for cell-replacement therapies and the treatment of human heart failure. The use of chemically defined media and small molecules that are GMP compatible permits the routine generation of millions of therapeutically applicable differentiated cardiomyocytes (CMs) from human embryonic stem cells (ESCs) [1]. The generation of patient-specific induced pluripotent stem cells (iPSCs) may overcome many of the immunological concerns associated with cell-based therapies, and recent reports of pharmacological elimination of hPSCs in differentiated cultures destined for transplantation, may have eliminated the *in vivo* tumorigenic potential of contaminating cells [2–4].

Among the small molecules critical for cardiomyogenesis, ascorbic acid (AA) has been recognized as a powerful inducer of CMs from both mouse and human PSCs [5–8]. Although the mechanism responsible for CM induction is unknown, mechanistically AA (or vitamin C) is known to promote collagen synthesis at the level of gene transcription and/or mRNA stability [9–11], and it is a critical co-factor for enzymatic hydroxylation of lysine and proline residues in pro-collagen [10,11]. Regulation of collagen biosynthesis [10] increases cardiac progenitor cell (CPC) proliferation via activation of the MEK/RTK-pathway [6,7]. High concentrations of AA, however, can have a negative biosynthetic effect on collagen types V and VI in cultured bovine aortic smooth muscle cells [12], and AA has a negative effect on cell-proliferation [13–15]. AA is an antioxidant [5], and it promotes ten-eleven translocation (Tet)-mediated generation of 5-hydroxymethylcytosine [16]. The latter can affect the DNA methylation state of mouse and human cells. Moreover, the effects of AA on CM differentiation require fibroblast growth factor (FGF)-receptors [8,17].

TGFβ-signaling through Activin/Nodal and BMP, as well as FGF and Wnt signaling, have been implicated in mesoderm induction and cardiac specification. Activin/Nodal and BMP ligands are members of the highly conserved TGFβ-superfamily [18], which signals through a heteromeric-complex composed of both serine/threonine kinase receptors (Type II) and Activin receptor–Like Kinases (ALKs, Type I). Upon ligand binding, Type II receptor dimers recruit and phosphorylate Type I receptors, which then initiate downstream signal-transduction cascades via phosphorylation of the Receptor (R)-SMADs (mothers against decapentaplegic homolog) proteins 1, 2, 3, 5, and 8/9. Type I receptors activation and subsequent phosphorylation of SMAD proteins is ligand specific with TGFβ/Activin/Nodal ligands activating ALK4, ALK5, and ALK7 to phosphorylate SMAD2 and SMAD3 (TGFβ-SMADs), while bone morphogenetic proteins (BMP)/growth differentiation factors (GDF) activate ALK2, ALK3, and ALK6 to phosphorylate SMAD1, SMAD5, and SMAD8 (BMP-SMADs). SMAD1 and SMAD5 can also be activated by ALK1, following TGFβ-engagement [19].

In mESCs, Activin/Nodal signaling is essential for proper commitment of mesendoderm [20], and Nodal-dependent TDGF1 (Cripto) activation promotes cardiomyocyte-induction by inhibiting neuronal differentiation [21]. The soluble BMP-antagonist Noggin has been reported to inhibit myocardial-differentiation of lateral mesendoderm-cultures *in vitro* [22], while BMP2-dependent SMAD-activation induces ectopic expression of myocardial-markers *in vivo*, and in anterior-lateral mesoderm-explant cultures, *in vitro* [22].

Since the first report by Takahashi et al [5], numerous groups have documented that AA promotes CM differentiation from both mouse and human ESCs and iPSCs [6,8,23,24]. AA is now considered indispensable for the routine production of hPSC-CMs using defined media [1]. Given the importance of BMP and TGFβ signaling in mesoderm induction and cardiac commitment, we hypothesized that AA may act through TGFβ ligand specification and differentiation pathways to promote cardiomyogenesis. To test this hypothesis, we examined the potential regulatory effects of AA on BMPs and Activin/Nodal/TGFβ signaling in differentiating mouse ESC lines. We find that AA modulation of BMP-SMAD signaling is critical to enhanced cardiomyogenesis via modulation of both SMAD1/5/8 and SMAD2/3.

## Material and methods

### Generation of cardiac restricted dsRED-positive mouse ESC lines

A plasmid carrying both the cardiac-restricted portion of the sodium-calcium exchanger 1 (*ncx1*) promoter, and a phosphoglycerate kinase promoter-driven neomycin resistant transgene (pGK-NeoR) was constructed. For tracking, the *ncx1* promoter (a fragment of 2730bp distal upstream region and 45bp of a cardiac-restricted untranslated exon [25]) was used to drive dsRED-Express1 protein. The pGK-NeoR-pA (1.7kb EcoRV/Sma1 fragment) from PGKneoKXRO was inserted into the multiple cloning site (EcoRI-BamHI digestion) in the reverse orientation, upstream of the *ncx1*-dsRED. The transgene construct was linearized and isolated by digestion with BamH1 and Nsi1, and 50 μg of plasmid were transfected into the mESC line R1 via electroporation, as previously described [26]. Positive (RFP) clones were selected with G418 (300 μg/ml, Gibco/BRL, Grand Island, NY), and the presence of both the *ncx1*-dsRed and *pgk*-NeoR sequences in the cell lines was confirmed by PCR. RFP-G418-resistant colonies were selected and tested for the expression of the pluripotency genes *Pou5f1-Oct3/4* and *Nanog*, and the lineage markers *Brachyury-T* (pan-mesoderm), *Sox1* (neuroectoderm), *Sox17* (pan-endoderm) and *Cdx2* (trophectoderm associated transcription factor). The expression of the mesoderm marker *Brachyury-T* was also validated in RFP-EBs at Day3/Day4 of differentiation, whereas the cardiac genes *Nkx2*.*5*, *GATA4* and *α-MHC* were assessed at Day 7+5 of differentiation, 48h after RFP6-CMs FACSorting (Day 7+3). Among the positive RFP-clones tested, clone RFP6-ESC was chosen to assess cardiac differentiation from mESCs. Data from all individual experiments were normalized to the maximum percentage of RFP^+^-cells, to minimize inter-experimental variability.

### Culture of ESCs and generation of ES-derived cardiomyocytes

The mESC line R1, its derived clone RFP6-ESCs, and the inducible cell lines iSMAD1 and iSMAD2 (kind gift of Dr. Todd Evans, Imperial Medical College, New York, NY, USA) were cultivated and differentiated as described [27–29]. Differentiation of ES cells into cardiomyocytes (CMs) was performed using the Hanging Drop (HD) technique through formation of Embryoid Bodies (EBs), using 300 cells/drop [27,29]. Seven days after formation, EBs were transferred to a 24-well plate for beating curve analysis or to 100 mm dishes for cell harvesting. Beating areas were counted daily as in indication of CM-differentiation. The stage of differentiation is reported as either Day *X* or Day *X*+*N*, where *X* refers to the time in suspension and *N* the number of days after plating. Cells were treated as described in the text with the following: Ascorbic Acid (1024M, Sigma, St. Louis, MO); TDGF1 (Cripto) and Activin A (both from R&D Systems, Minneapolis, MN); BMP2 (R&D Systems); Vitamin E and N-Acetyl-Cysteine (NAC, both from Sigma, St.Louis, MO); Doxycycline (Gibco/BRL); SB431542 (Tocris Bioscience, Minneapolis, MN); and Dorsomorphin (EMD Millipore, Billerica, CA). Specifically, treatments were performed as a single dose at the following stages of differentiation: Day 0 (dissociated cells, at the time of EB formation); Day 2 (following washing of EBs) and Day 5 (EBs in suspension). Once begun, treatments were continued throughout the duration of the experiment until cells were harvested for analysis, with the exception of the doxycyline (Dox) treatment, which was performed for 24 hours, beginning on Day 2 and ending on Day 3. On Day 3, medium was changed to stop Dox treatment. Cells were never refreshed.

### RT, PCR and qPCR

Total RNA was extracted using Trizol (Invitrogen) followed by DNAse treatment. RNA abundance was determined after Reverse Transcription (RT) and either standard or quantitative PCR (qPCR) techniques [30]. cDNA synthesis was performed using 500 ng of total RNA with a High Capacity cDNA Archive Kit (Applied Biosystems/Life Technologies, Grand Island, NY). PCR reactions were performed with the GeneAmp PCR System 9700 using the AmpliTaq Gold DNA Polymerase (both Applied Biosystems). Quantitative PCR reactions were performed with the ABI PRISM 7900HT Sequence Detector System using the core reagent kit and the SYBR Green PCR Master Mix (all Applied Biosystems) in a 384 well plate format. Primers sequences have been previously described [26].

### Immunostaining

R1- and RFP6-derived CMs were examined by immunostaining using primary antibodies to α-actinin (Sigma, 1:800, A7811) and TNNT2 (NeoMarkers, 5 μg/mL, MS295R7). Cells were fixed in 2% paraformaldehyde in PBS, washed (3x with PBS) and permeabilized (0.2% Triton X-100, Sigma). Non-specific binding was blocked with a solution of 1% bovine serum albumin (BSA) in PBS. AlexaFluor 488- or 568-conjugated goat anti-mouse IgG or AlexaFluor 568-conjugated goat anti-rabbit IgG (all Invitrogen) were used as secondary antibodies (1:1000). Nuclei were stained with Hoechst 33342 (Molecular Probes, Eugene, OR, 5 mg/mL). Images were obtained by fluorescence microscopy using a Zeiss Axiovert 35 microscope (Zeiss, West Germany) with Zeiss lenses (Plan-Neofluar, 63X/1.25 oil, 40X/1.30, 10X/0.25 and 5X/015) coupled to a SPOT Camera (Diagnostic Instruments, Inc Sterling Heights, MI). Following acquisition (SPOT Advanced 4.0.9 software), single channel files (tiff or jpeg) were composed with the assistance of Photoshop and the adjustment of only contrast or brightness.

### Cell cycle analysis

EBs at Day 3 and Day 4 of differentiation were trypsinized, and single cells in suspension were fixed with an ice-cold solution of methanol/acetone (1:1). The DNA content was measured on propidium iodide (PI, Sigma) stained nuclei using a FACSCanto (BD Biosciences, San Jose, CA), as previously described [30]. Cell cycle compartments were deconvoluted from single-parameter DNA histograms of 10,000 cells.

### Western blotting

Proteins (10–25 μg) from cell lysates were separated by SDS-PAGE, transferred to PVDF membranes (BioRad, Hercules, CA) and non-specific sites blocked with non-fat dry milk (Sigma, M7409). Blots were probed with the following primary antibodies: rabbit pSMAD2 (1:1000, Millipore, Billerica, MA AB3849); rabbit SMAD2/3 (1:1000, Cell Signaling, Danvers, MA 3102); rabbit pSMAD1/5/8 (1:1000, Cell Signaling 9511); mouse SMAD1 (1:2000, Santa Cruz, Dallas, TX sc-913C1b); goat T (or Brachyury) (1:500, Santa Cruz sc-17745); and goat or mouse GATA4 (both from Santa Cruz, 1:500 sc-1237, or 1:250 sc-25310, respectively). Horseradish peroxidase (HRP)-conjugated goat anti-rabbit IgG (H+L), goat anti-mouse IgG (H+L) or rabbit anti-goat IgG (H+L) (Zymed/Life Technologies Grand Island, NY) were used as the secondary antibodies. HRP was detected using Pierce Super Signal ECL substrate kit (Pierce/Thermo Scientific Rockford, IL). Chemiluminescence was captured on film (Phenix Research Products, Candler, NC), and densitometry analysis was performed using Kodak Molecular Imaging (MI) software (Carestream Health, Inc, Rochester, NY). Amido Black (Sigma) staining of total protein was used as control for total protein loading.

### Flow cytometry

For cardiac differentiation assessment, RFP6-, iSMAD1- and iSMAD2-EBs were harvested at Day 7+3. For the RFP6-ESC clonal cell line, flow cytometry analysis was performed on single cell suspension of live cells, and cardiomyogenesis quantified by the amount of RFP protein. For the inducible clonal cell lines iSMAD1-ESCs and iSMAD2-ESCs, following trypsinization, single-cells were fixed with Fix/Perm buffer (BD Biosciences) and stained with the primary mouse antibody to TNNT2 (NeoMarkers MS295R7), followed by incubation with a secondary AlexaFluor 647 conjugated chicken anti-mouse IgG (Life Technologies). A mouse-IgG1 antibody was used as the isotype control (Life Technologies MG100). All data were acquired using a FACSCanto (BD Biosciences), counting a minimum of 5000 to 10,000 events.

### Statistical analysis

Results are presented as mean ± SEM. Paired Student’s *t*-test was employed to determine statistical significance. P<0.05 was considered statistically significant.

## Results

### Ascorbic acid’s cardiogenic potential is stage- and dose-dependent

Cardiomyocytes (CMs) were differentiated from the mESC clonal line RFP6, harboring a dsRED transgene encoding the red fluorescence protein (RFP) under the control of the distal upstream cardiac restricted portion of the sodium-calcium exchange 1 gene (*ncx1*) promoter. This promoter is cardiac restricted during embryonic and fetal development [25], and confers the highest degree of cardiac specificity compared to other cardiac specific genes [31,32], being present only in fully committed atrial, ventricular and conduction system associated cardiomyocytes. It is uniformly expressed in the heart starting in the cardiac progenitors containing the cardiogenic plate region at 7.75dpc [25]. Similar to the parental R1 mESC-line, RFP6-ESCs differentiated into spontaneously contracting cells that could be observed routinely on Day 7+1. At Day 7+3, beating cell clusters could be observed in >90% of the plated EBs, a frequency that was almost identical to that seen with the parental R1 line. When examined by flow cytometry, 0.03–0.05% of the cells evaluated at Day 7+1 were positive for RFP. This frequency increased to 0.5% of total cells at Day 7+3, consistent with the ~0.35% TNNT2^+^-CMs observed (not shown) in the syNP4 mES-clonal cell line, with a (*ncx1*) promoter driven puromycin resistance cassette [26]. Up to 1.5% of untreated RFP^+^-CMs at Days 7+5 to 8 were positive for dsRED. Sorted and re-plated RFP^+^-CMs were tested for TNNT2 expression; positivity confirmed that they corresponded to CMs. For additional clonal line characterizations, see S1 Fig.

**Fig 1:**
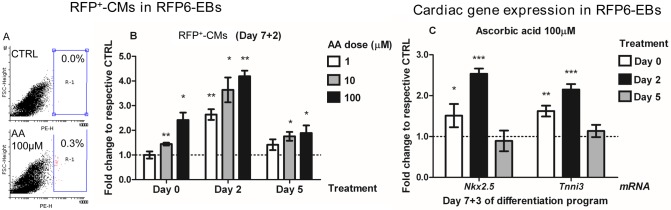
Ascorbic acid promotes cardiomyogenesis in a stage-specific and a dose-dependent manner. **A.** Flow cytometry analysis of RFP fluorescence in differentiating RFP6 Embryoid Bodies (EBs) at Day 7+2, in AA-treated EBs (Day 2), compared to untreated controls. EBs were plated at Day 7 and fluorescence could be observed as early as Day 7+1. **B.** RFP6-ESCs (Day 0, n = 3) and RFP6-EBs (Day 2, Day 5, n = 3) were treated with various doses of AA (1 μM, 10 μM, 100 μM) and analyzed for RFP fluorescence at Day 7+2 of differentiation. **C.** Quantitative PCR analysis of cardiac markers in RFP6-EBs at Day 7+3 of differentiation, after treatment with AA (100 μM) at Day 2 (n = 3). *p<0.05, **p<0.01 and ***p<0.001 vs untreated control.

**Fig 2:**
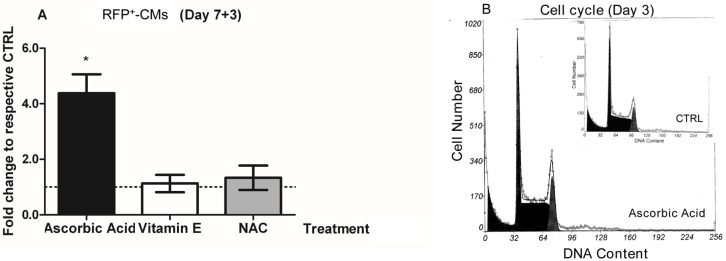
Ascorbic acid-mediated cardiogenic-induction is independent of its anti-oxidant and anti-proliferative capacities. **A.** Cardiomyogenesis (RFP fluorescence) assessed in RFP6-EBs at Day 7+3 after treatment with AA (100 μM), Vitamin E (100 μM) and NAC (1000 μM) (n = 3) performed at Day 2 of differentiation. **B.** Analysis of DNA content (PI staining) of dissociated EBs at Day 3 of differentiation, 24 hours after treatment with AA (Day 2, 1000 μM, n = 3). The histograms show cell cycle stages G0/G1, S and G2/M. No difference in the percentage of cells could be demonstrated between the treated and untreated controls. *p<0.05 compared to untreated control.

We employed the RFP6-ES clonal line to determine the effects of AA (ranging in concentration from 1 to 100 μM and including the physiologic dose of 70–80 μM) [33,34] on cardiomyogenesis at three stages of differentiation: Day 0, Day 2 and Day 5. The optimal concentrations for inducing RFP were found to be 10 or 100 μM. Relative to controls and compared to the earliest stages of differentiation (Day 7 to 7+2), when the number of CMs is low, the number of RFP^+^-cells (RFP^+^-CMs) was increased significantly by 2.5-fold (p = 0.01) when AA was added at Day 0, by 4.2-fold (p = 0.0001) when AA was added at Day 2, and by 1.9-fold (p = 0.04) when AA was added at Day 5 (Fig 1A and 1B), showing that AA-treatment at Day 2 produced the greatest effect. The simultaneous and significant increase in cardiac transcripts for *Nkx2*.*5* (2.6-fold, p<0.0001) and cardiac-Troponin I (*Tnni3*, 2.2-fold, p = 0.009) at Day 7+3, together with the increased numbers of RFP^+^-CMs, confirmed the ability of AA to induce cardiomyogenesis in RFP6-ESCs when added at Day 2 of differentiation (Fig 1C). When we treated the RFP6-EBs at Day 2 with the antioxidants Vitamin E (VitE) and N-Acetyl-Cysteine (NAC), we were unable to demonstrate any significant effect on the number of RFP^+^-CMs at Day 7+3 (Fig 2A). Cell-cycle progression performed at Day 3 was also unaffected by AA-treatment (Day 2) at all doses tested (100 μM, not shown, and 1000 μM, Fig 2B). These results demonstrate that AA enhances *ncx1*-driven dsRED transgene expression in a concentration- and time-dependent manner, and that induction of cardiomyogenesis is not significantly related to the antioxidant function of AA or its potential anti-proliferative capacity.

### Active but not total SMAD proteins are modulated by ascorbic acid

**Fig 3:**
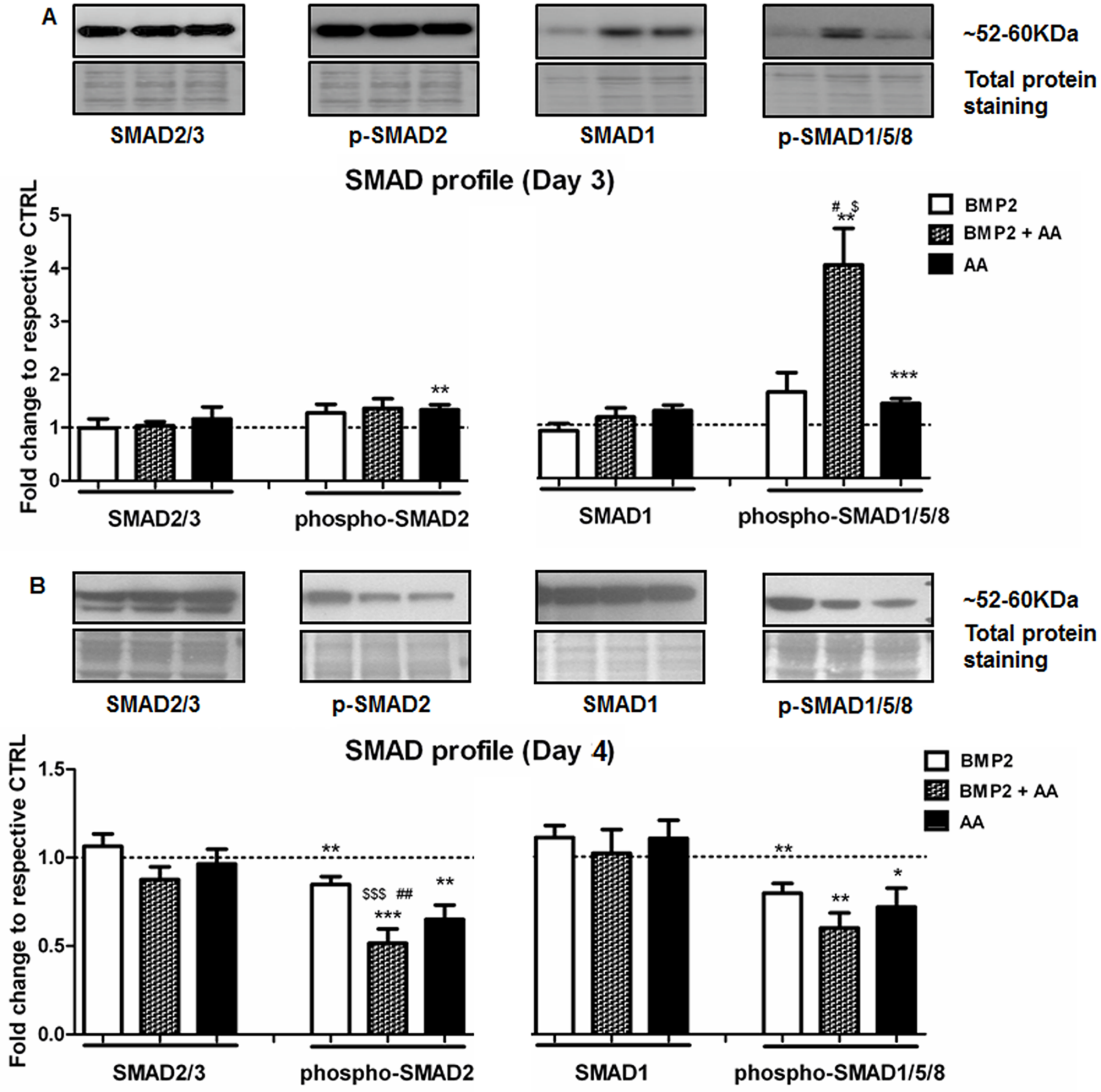
Ascorbic acid-enhanced cardiomyogenesis is SMAD-modulation dependent. Western Blots of total- and active-SMADs at Day 3 (**A**) and Day 4 (**B**) of the differentiation process, after treatments with BMP2 (Day 0), AA (Day 2) and their combination (Day 0 + Day 2). **A.** Total-SMAD2/3 (n = 6), phospho-SMAD2 (n = 14), total-SMAD1 (n = 6) and phospho-SMAD1/5/8 (n = 16); **B.** Total-SMAD2/3 and phospho-SMAD2 (n = 8) and total-SMAD1 and phospho-SMAD1/5/8 (n = 10). *p<0.05, **p<0.001 and ***p<0.0001 are relative to untreated control; ^$^p<0.05 and ^$ $ $^p<0.001 relative to BMP2 treatment; ^#^p<0.05 and ^##^p<0.01 relative to AA treatment.

To determine whether AA modulates BMP- and TGFβ/Activin/Nodal-signaling via SMAD activation, we assessed total SMAD (SMAD2/3 and SMAD1), and activated (phosphorylated) SMAD-protein (p-SMAD2 and p-SMAD1/5/8) abundance in differentiating R1 mESCs (Fig 3). Cells were treated with BMP2 (Day 0), AA (Day 2) or their combination (Day 0 + Day2), and both total- and phospho-SMADs were examined at two time points (Day 3 and Day 4 of differentiation). At Day 3 of differentiation, no change in total proteins SMAD2/3 or SMAD1 was observed after any treatment, relative to untreated controls. In contrast, AA modestly increased the level of phospho-SMAD2 (1.34-fold, p = 0.004) and phospho-SMAD1/5/8 (1.41-fold, p = 0.004)(Fig 3), whereas BMP2 tended to non-significantly reduce total SMAD1. The combined treatment of BMP2 and AA, however, synergistically, selectively, and very significantly increased the phosphorylation of SMAD1/5/8 (4.01-fold, p = 0.007) relative to untreated controls. Cardiac and mesodermal proteins GATA4 (mesendoderm) and T protein (pan-mesoderm), were also significantly increased at Day 3 of differentiation (S2 Fig) by the addition of AA (1.44-fold, p = 0.03; 1.35-fold, p = 0.008; respectively). Consistently at Day 4 of differentiation, no change in total SMAD-proteins could be demonstrated under any condition (Fig 3B). At this latter time point, the quantity of activated SMADs was significantly reduced by all treatments in contrast to what was observed at Day 3. Relative to controls, phospho-SMAD2 was significantly reduced by 25% following BMP2-addition, 30% after AA-treatment, and by 40% following the combined treatment of AA and BMP2 (p = 0.0002 vs untreated control, p = 0.0003 vs BMP2 and p = 0.002 vs AA) (Fig 3B). Phospho-SMAD1/5/8 was also significantly decreased by 20% after BMP2-treatment, 35% following AA-induction, and 50% after co-treatment with AA and BMP2 versus untreated control (Fig 3C). These latter data suggest that the phospho-SMAD ratio is tightly regulated during cardiomyogenesis, but that the combined treatment of the cells with both AA and BMP2 can cause a transient and highly significant increase in phospho-SMAD1/5/8.

### Ascorbic acid induction of cardiomyogenesis involves SMAD signaling

**Fig 4:**
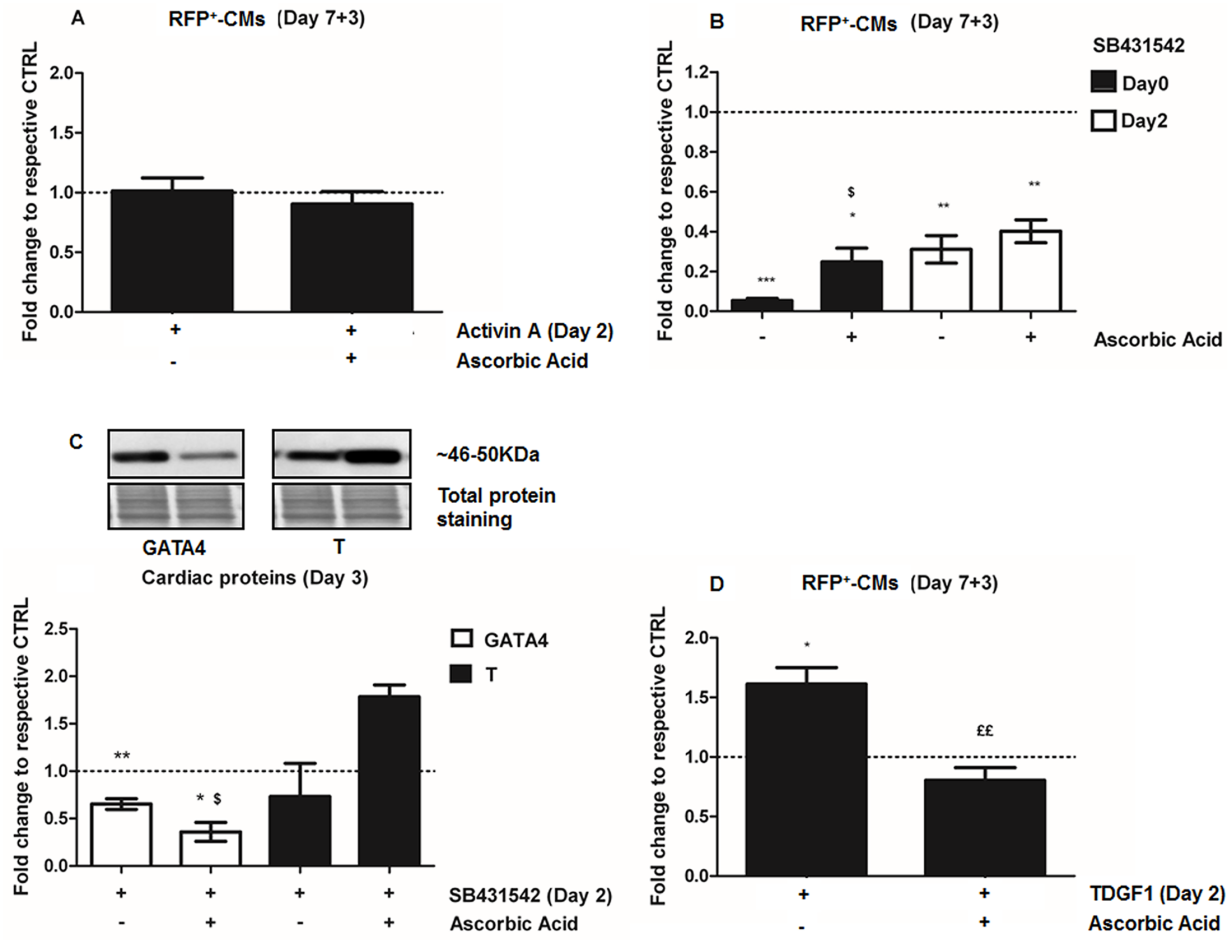
Ascorbic acid induction of cardiomyogenesis involves the TGFβ-signaling pathway. **A-B.** Cardiomyocyte-induction assessed by flow cytometry of RFP^+^-CMs at Day 7+3 of differentiation following treatments with **A.** Activin A (100 ng/mL) added at Day 2 (n = 12) and **B.** SB431542 (10 μM), an inhibitor of SMAD2-activation, added at Day 0 (n = 3) or Day 2 (n = 3), performed alone or in combination with AA (Day 2). **C.** Western Blot of the cardiac markers GATA4 and T assessed at Day 3 of differentiation following treatments with SB431542 (10 μM) and AA, both performed at Day 2 (n = 4). **D.** Flow cytometry assessment of cardiomyogenesis (RFP^+^-CMs) at Day 7+3 of differentiation following treatments with TDGF1 (100 ng/mL) added at Day 2 (n = 3), alone or in combination with AA (Day 2).*p<0.05, **p<0.01, ***p<0.0001 are compared to untreated control; ^$^p<0.01 compared to SB431542 inhibitor; ^££^p<0.01 compared to TDGF1 treatment.

To determine how AA induction of CMs from mESCs modulated SMAD signaling and cardiac induction in response to Type II receptors, we incubated RFP6-EBs with pharmacological agents that modulate TGFβ superfamily agonists [21,35–37]. The TGFβ superfamily activator Activin-A, alone or in combination with AA, did not have any significant effect on the number of RFP6-CMs (RFP^+^-CMs) (Fig 4A). Addition of SB431542, a selective inhibitor of activin receptor like kinases ALK4, ALK5 and ALK7, which has been shown to inhibit cardiomyogenesis when added during the early phase of differentiation (either Day 0 or Day 2) [38], prevented TGFβ-SMAD2 and SMAD3 activation/phosphorylation, as expected, demonstrating a stage- and time-dependent reduction in RFP^+^-CMs. Specifically, the number of RFP^+^-CMs decreased by 20-fold (p = 0.0001) when SB431542 was added alone at Day 0, and by 3.3-fold when inhibition performed at Day 2 (p = 0.002 vs untreated control, p = 0.03 vs SB431542-treatment at Day 0) (Fig 4B). AA was able to rescue, at least partially, the decrease in RFP^+^-CMs following SB431542-treatment, but only when inhibition was performed at Day 0 (p = 0.04 vs SB431542-treatment alone) (Fig 4B). In Day 3 RFP6-EBs, SB431542 significantly down-regulated GATA4 (p = 0.002), but not the mesodermal marker protein T (Fig 4C). Addition of AA at Day 2 of differentiation to SB431542 treated RFP6-EBs further reduced GATA4 (0.36-fold, p = 0.03 vs SB41542) but the increase in T was not significant (Fig 4C). These data suggest that AA affects mESC differentiation during early cardiac specification.

The nodal co-receptor TDGF1 (Cripto) is the prototypic member of the EGF-CFC (epidermal growth factor-like-cripto-FRL-1-cryptic) family of EGF-like molecules, and it is critical for the transition from myocardial specification to differentiation [39]. When TDGF1 (100 ng/ml) was added to RFP6-EBs at Day 2 (p<0.05) of differentiation, the number of RFP^+^-CMs significantly increased. No significant effect was observed when it was added at Day 0 (not shown). AA, when added alone at Day 2, also led to a 1.25-fold induction (p = 0.002) of TDGF1 at Day 3 of differentiation (S2 Fig). However, when TDGF1 and AA were both added at Day 2 of differentiation, the number of RFP^+^-CMs at Day 7+3 significantly decreased (p = 0.005) relative to TDGF1-induction (Fig 4D). These data are consistent with an effect of AA to modulate pathways during the specification to differentiation transition normally mediated by TDGF1.

**Fig 5:**
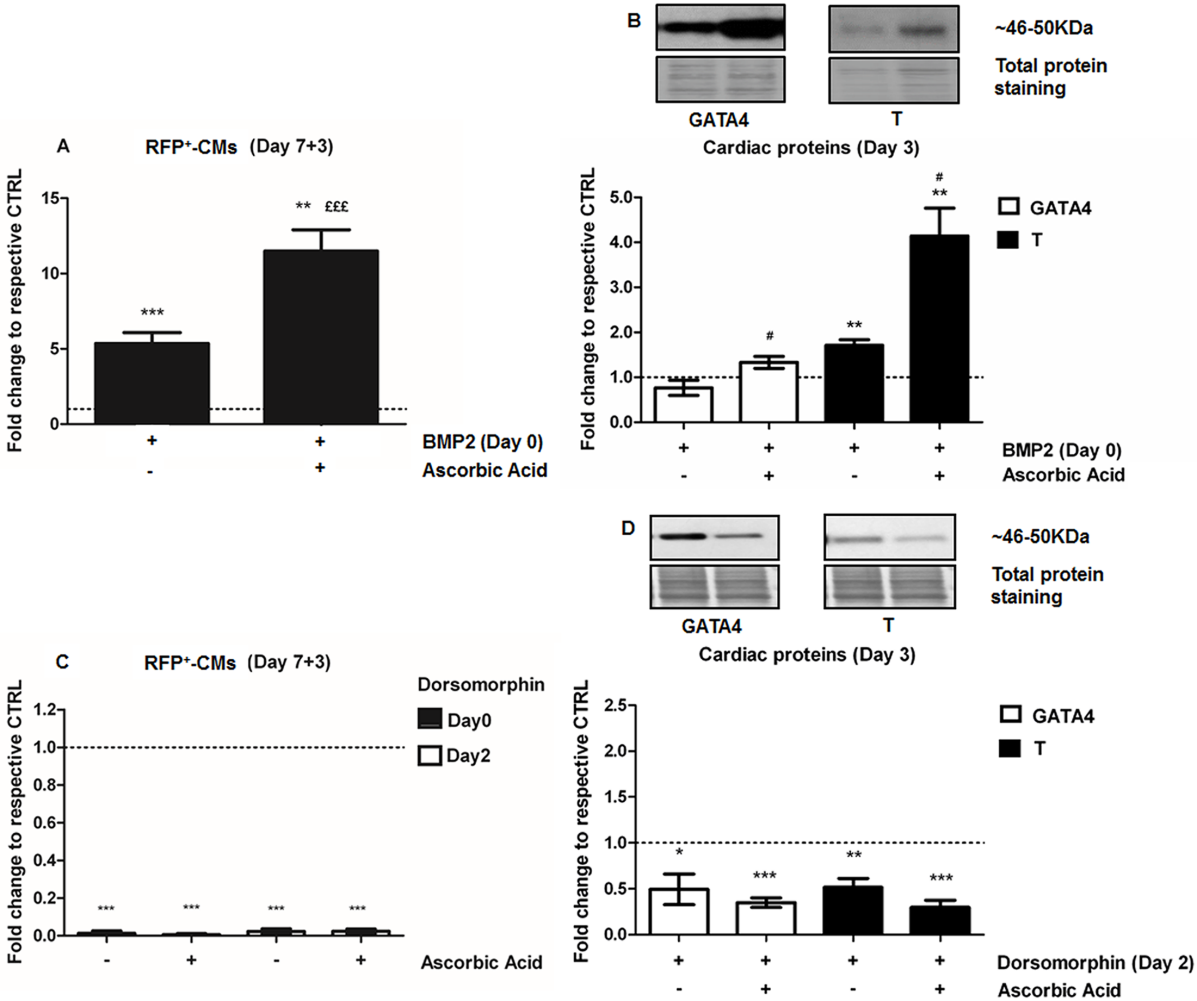
Ascorbic acid induction of cardiomyogenesis involves the BMP-signaling cascade. **A.** Cardiomyogenesis assessment by quantification of RFP^+^-CMs by flow cytometry analysis after treatment with BMP2 (10 ng/mL), added at Day 0, Ascorbic acid, added at Day 2, and their combination (Day 0 + Day 2, n = 5). **B.** Western Blots of the cardiac markers GATA4 and T following treatment with BMP2 (10 ng/mL, Day 0), AA (Day 2), and their combination (Day 0 + Day 2, n = 6). **C.** Cardiomyogenesis assessment by flow cytometry quantification of RFP^+^-CMs after dorsomorphin treatment (2 μM), an inhibitor of SMAD1-activation, added at Day 0 or Day 2, alone or in combination with AA (Day 2) (n = 3). **D.** Western Blots of the cardiac markers GATA4 and T following dorsomorphin inhibition (Day 2, 2 μM, n = 5), alone or in combination with AA (Day 2). *p<0.05, **p<0.01 and ***p<0.001 are relative to untreated control; ^£££^p<0.001 relative to BMP2 treatment, ^#^p<0.05 relative to AA treatment.

Stimulation of BMP/GDF pathway by BMP2 at Day 0 resulted in the numbers of RFP^+^-CMs being significantly increased at Day 7+3 (6.4-fold, p = 0.0002) compared to untreated control (Fig 5A), but when both BMP2 (Day 0) and AA (Day 2) were added together, RFP^+^-CMs increased by 12-fold (p = 0.002). To better assess how AA functions to promote cardiomyogenesis, we examined the effects of AA in the presence of BMP2 or dorsomorphin, an inhibitor of BMP signaling that selectively inhibits the BMP type I receptors ALK2, ALK3 and ALK6 to block BMP-mediated SMAD1/5/8 phosphorylation [40], but not AMP-activated kinase activity [41]. At the protein level, BMP2 alone significantly up-regulated T protein (1.71-fold, p = 0.005) (Fig 5B), but did not change GATA4 abundance at Day 3 of differentiation. When BMP treatment was combined with AA, both GATA4 and T proteins were significantly up-regulated (1.33-fold, p = 0.05; 4.14-fold, p = 0.007; respectively) (Fig 5B). In contrast to BMP2, dorsomorphin, severely impaired the generation of RFP^+^-CMs at both Day 0 and Day 2 of differentiation (Fig 5C). AA-treatment was unable to rescue the cardiac-program following treatment with dorsomorphin (Fig 5C), indicating that BMP signaling is essential for cardiomyogenesis. The abundances of GATA4 and T were also reduced significantly in EBs at Day 3, 24 hours after treatment with dorsomorphin (Fig 5D). These data demonstrate that active BMP signaling, most likely involving SMAD1/5/8 phosphorylation, is required for cardiomyocyte differentiation and that AA acts synergistically with the BMP signaling pathway to promote CM formation.

### Ascorbic acid enhances cardiomyogenesis acting in concert with SMAD1

To delineate how AA interacts with BMP/GDF- versus Activin/Nodal-mediated SMAD activation, we used the inducible (i) iSMAD1- and iSMAD2-mESC clonal lines. This conditional model system permitted the control of SMAD1 or SMAD2 transgene expression during EB differentiation [42]. Upon stimulation of the tetracycline (tet) operator by doxycycline (Dox), SMAD(1 or 2) upregulation could be visualized by GFP expression. Moreover, when Dox was removed from the medium, SMAD(1 or 2) returned to basal level (not shown). In these experiments, we performed SMAD(1 or 2) induction for 24 hours, by adding Dox at Day 2 of differentiation, and removing it at Day 3 of differentiation by changing the medium. Under these conditions, GFP and, by extension, SMAD(1 or 2) were present within 6 hours after addition of Dox in differentiating EBs (not shown).

**Fig 6:**
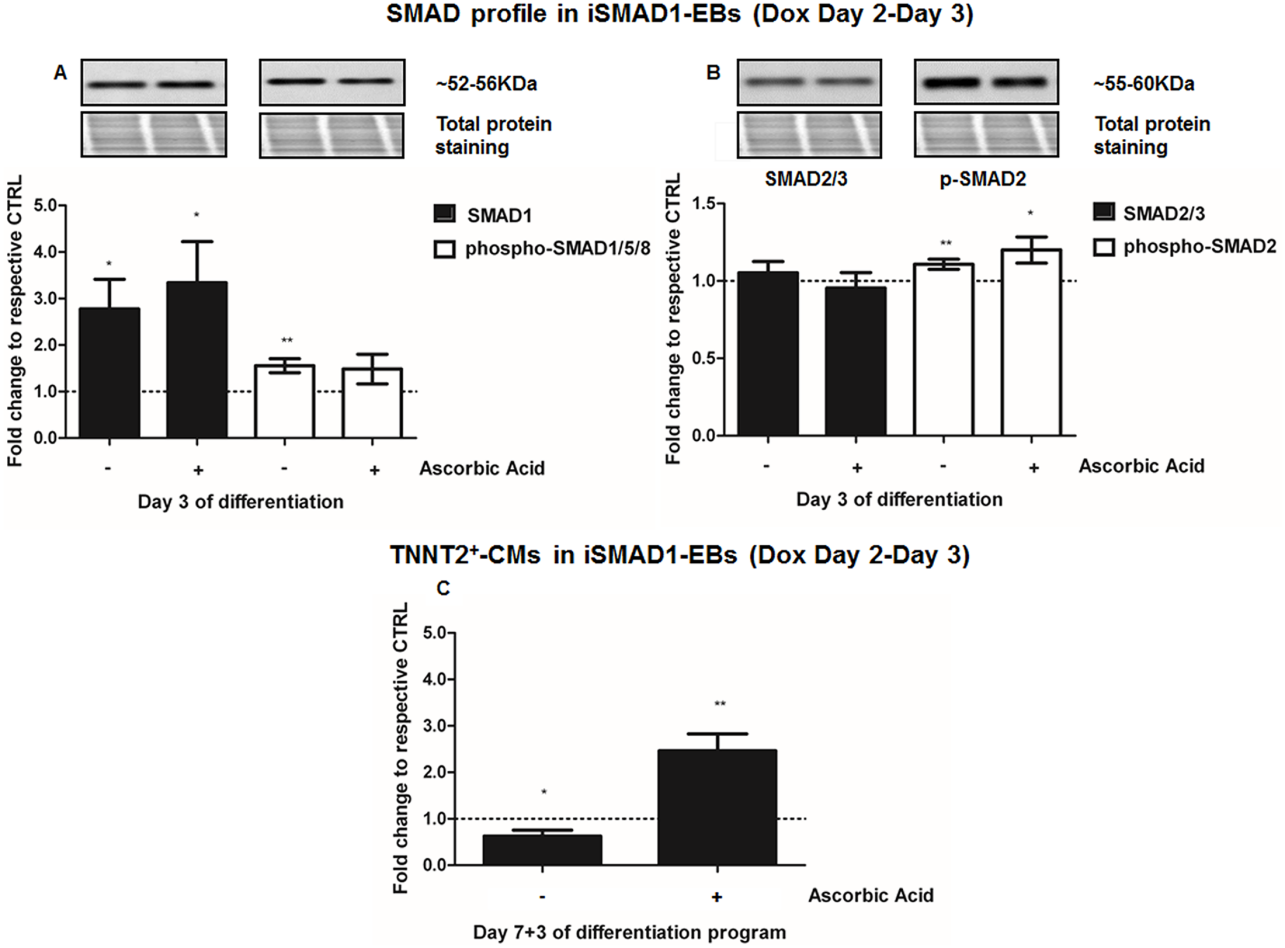
Ascorbic acid’s cardiac potential is potentiated by SMAD1. **A-B.** Western Blot at Day 3 of differentiation of **A.** total-SMAD1 and phospho-SMAD1/5/8, and **B.** total-SMAD2/3 and phopsho-SMAD2 in iSMAD1-EBs after induction with doxycycline (Dox, 1 μg/mL, for 24 hours from Day 2 to Day 3), AA induction (Day 2) and their combination, compared to untreated control (iSMAD1-mESCs, n = 6). **C.** Cardiomyogenesis assessment by flow cytometry quantification of TNNT2^+^-CMs in iSMAD1-EBs at Day 7+3 of differentiation following SMAD1-conditional stimulation by doxycycline (Dox) treatment performed for 24 hours, from Day 2 to Day 3 of the differentiation program, AA induction (Day 2) and their combination, compared to untreated control (iSMAD1-EBss, n = 5). *p<0.05 and **p<0.01 compared to untreated iSMAD1-EBs.

**Fig 7:**
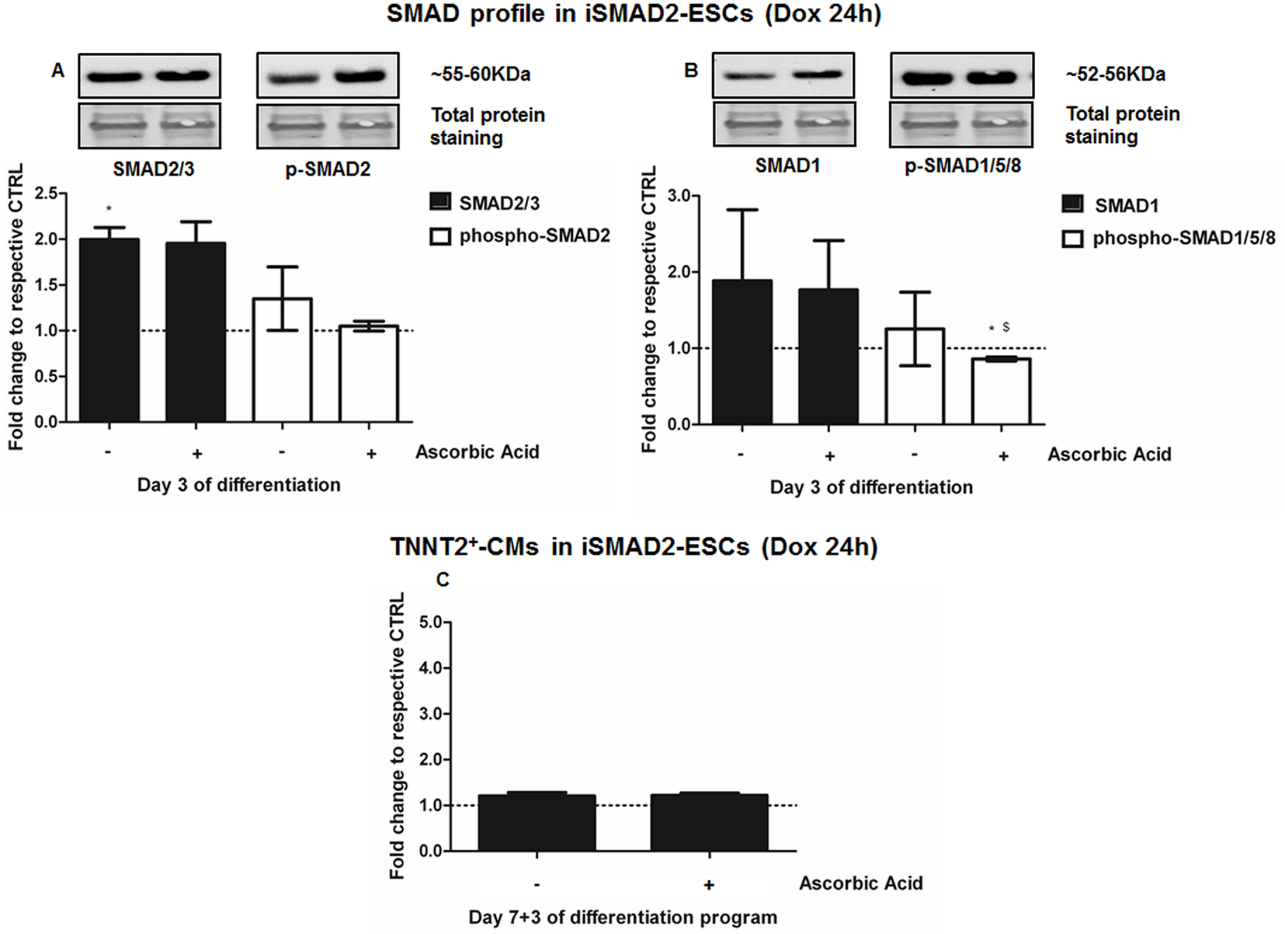
Ascorbic acid’s cardiac potential is antagonized by SMAD2. **A-B.** Western Blot at Day 3 of differentiation of **A.** total-SMAD2/3 and phospho-SMAD2 and **B.** total-SMAD1 and phospho-SMAD1/5/8 proteins in iSMAD2-EBs after induction with doxycycline (1 μg/mL, 24h from Day 2 to Day 3), AA induction (Day 2) and their combination, compared to untreated control (iSMAD2-EBs, n = 3). **C.** Cardiomyogenesis assessment by flow cytometry quantification of TNNT2^+^-CMs in iSMAD2-EBs at Day 7+3 of differentiation following SMAD2-induction with doxycycline (Dox) for 24 hours, from Day 2 to Day 3 of differentiation, AA induction (Day 2) and their combination, compared to untreated control (iSMAD2-EBs, n = 3). *p<0.05 compared to untreated control; ^$^p<0.05 compared to Dox.

We performed protein assessment at Day 3 of differentiation, 24 hours after addition of Dox and AA, alone or in combination, to iSMAD1-ESCs (iSMAD1-EBs, Fig 6) or to iSMAD2-ESCs (iSMAD-EBs, Fig 7). In these experiments, Dox-induced SMAD1 over-expression increased total-SMAD1 by 2.8-fold (p = 0.04)(Fig 6A), while total-SMAD2 showed no significant change (Fig 6B). In addition, phospho-SMAD1/5/8 and phospho-SMAD2 also increased by 1.56-fold (p = 0.01)(Fig 6A), and 1.11-fold (p = 0.02)(Fig 6B), respectively. When SMAD1 was overexpressed by Dox-treatment (Day 2 to Day 3), AA further increased total SMAD1 by up to 3.34-fold (p = 0.04)(Fig 6A) and phosho-SMAD2 by 1.2-fold (p = 0.05)(Fig 6B), but no further increase could be demonstrated for phospho-SMAD1/5/8 (Fig 6A). The addition of AA (Day 2) to SMAD1 overexpressing cells significantly increased the number of TNNT2^+^-CMs (2.5-fold, p = 0.003 vs untreated controls = iSMAD-CMs, p = 0.002 vs SMAD1-induction) (Fig 6C). AA also significantly increased GATA4 (p = 0.04) and T protein (p = 0.002) abundance in iSMAD1-EBs, relative to untreated iSMAD-EBs (S3A Fig).

In iSMAD2-ESCs, Dox-treatment on iSMAD2-EBs from Day 2 to Day 3 increased total SMAD2/3 abundance by 2.0-fold (p = 0.02) at Day 3 of differentiation (Fig 7A). However, it did not have any significant effect on phospho-SMAD2 (Fig 7A), total SMAD1 or phospho-SMAD1/5/8 (Fig 7B), relative to uninduced iSMAD2-EBs. Addition of AA (Day 2) with SMAD2-induction by Dox (Day 2 to Day 3) on iSMAD2-EBs did not have any effect on total SMADs or phospho-SMAD2 (Fig 7A and 7B), but it reduced phospho-SMAD1/5/8 level (0.86-fold, p = 0.04 vs untreated iSMAD2-EBs and p = 0.03 vs SMAD2-induction) (Fig 7B). Neither SMAD2 overexpression by Dox nor AA-treatment significantly changed the total number of TNNT2^+^-CMs at Day 7+3 of differentiation, compared to untreated control (Fig 7C). No effects on GATA4 or T proteins were observed at any time point examined (S3B Fig) in SMAD2 overexpressing cells. The data shown in Figs 6 and 7 thus implicate SMAD1 in the up-regulation of cardiomyocytes caused by AA.

## Discussion

Although the mechanism responsible for CM-induction is incompletely understood, mechanistically AA (or vitamin C) is known to promote collagen synthesis at the level of gene transcription and/or mRNA stability [9–11], and it is a critical co-factor for enzymatic hydroxylation of lysine and proline residues in pro-collagen [10,11]. Regulation of collagen biosynthesis [10] increases cardiac progenitor cell (CPC) proliferation via activation of the MEK/RTK-pathway [6,7]. Despite the importance of AA to cardiomyogenesis, its mechanism of action has remained enigmatic. This study has revealed a new mechanism whereby AA affects mESC differentiation. AA specifically enhances the differentiation of mESCs to CMs in a highly reproducible manner that is sensitive to SMAD1 blockade, and which changes the ratios of active SMAD proteins to modulate the differentiation process. Specifically, we provide evidence that AA modulates the differentiation of PSCs to CMs by quantitatively altering phosphorylated forms of SMAD2/3 and SMAD1/5/8 without changing total SMAD protein content. This modulation leads to altered ratios of active SMADs, which when disrupted, can either promote or prevent cardiomyogenesis.

Our study took advantage of genetically modified mESCs (RFP6-ESCs, iSMAD(1 or 2)-ESCs) and pharmacological interventions (Activin A, TDGF1, BMP2, SB431542 and dorsomorphin) to unravel the role of AA and SMAD signaling. A mouse ESC-clonal line that express the dsRED transgene (RFP protein) only in CMs (RFP6-ESCs) permitted the quantification of cardiac cells throughout the differentiation process, whereas two mESC-clonal lines with conditional (inducible) expression of either SMAD1 or SMAD2 by doxycycline, allowed transient SMAD-manipulation at key-stages (Day 2 to Day 3) of differentiation. As expected and consistent with prior reports [5,6,8], AA alone increased the numbers of CMs (RFP^+^-CMs) in a dose and time-dependent manner. When BMP2, which induces mesoderm formation, was administered at Days 0 in combination with AA (Day 2), the result was an increased SMAD phosphorylation and a synergistic amplification of CM production (RFP^+^-CMs). Conditional overexpression of SMAD1 coupled with treatment by AA also led to a significant increase in CM numbers (TNNT2^+^-cells). GATA4 and T proteins were both increased following AA-treatment, alone or in combination with BMP2 or SMAD1-induction.

Inhibition of SMAD-kinases ALK1/2/3/6 by dorsomorphin completely blocked the differentiation of mESCs to CMs, while inhibition of ALK4/5/7 by SB431542 only partially blocked this process. AA could antagonize some of the inhibitory effects caused by SB431542 but had no effect following dorsomorphin inhibition. It thus appears that activation of the BMP-cascade is necessary and sufficient for the cardiac-process to occur, whereas inhibition of TGFβ-signaling by SB431542 can be compensated in part by the addition of AA. At the protein level, inhibition by SB431542 affected both TGFβ-dependent SMAD2 and SMAD1/5 activation [43]; whereas, dorsomorphin was selective for the BMP-SMADs. We suggest that the SB431542-dependent reduction in phospho-SMAD1/5/8 is a consequence of the reduced phospho-SMAD2 levels. In fact, the TGFβ-lateral (non-canonical) signaling, which is propagated through ALK1-SMAD1/5, is ALK5-dependent. Therefore, a non-functional TGFβ-canonical (ALK5) pathway, may negatively affect the TGFβ-lateral (ALK1) pathway, possibly during the assembling of the hetero-complex TGFβ/ALK5/ALK1 [19]. Therefore, it appears that AA has a negative effect on TGFβ-SMAD1/5 activation, which is readily visible only by blocking BMP-dependent SMAD1/5/8 activation. Since the activation of the TGFβ-parallel pathway is less sustained compared to the canonical pathway, we further speculate that the positive effect of AA on cardiomyogenesis relies on the simultaneous modulation of these two TGFβ-signaling pathways.

Embryonic signals associated with mesoderm induction involve Wnt pathway stimulation, and activin/nodal, BMP and FGF cascades [17,21,44–48], while embryonic signals associated with cardiac specification involve Wnt inhibition, and BMP and TGFβ signaling. Like Wnt signaling, the effects of TGFβ signaling (specifically SMAD2) on mESC-cardiac differentiation are bi-modal [38]. Nodal/TDGF-dependent SMAD2-activation is pro-cardiac in the early phases of the cardiac program (mesoderm induction and cardiac specification), while TGFβ-dependent SMAD2-phosphorylation at later stages (Day 5) inhibits CM-proliferation and differentiation [38]. Addition of AA at Day 0 of differentiation had no effect on cardiomyogenesis or on Activin A signaling; instead, the major effects of AA were observed when it was applied at Day 2 of differentiation. Treatment of differentiating mESCs stimulated the production of TDGF1 protein by Day 3, but addition of AA had competing effects with exogenously added TDGF1 during the transition from mesoderm induction to early cardiac specification (Day 2 to Day 3). We interpret these findings as evidence that AA contributes to cardiac specification and differentiation, and not mesoderm induction, through modulation of SMAD signaling caused by both BMP/GDF and Activin/Nodal receptor-mediated activation.

Receptor-regulated SMAD-proteins (R-SMADs) are a category of SMADs activated by phosphorylation upon receptor recruitment, following ligand stimulation. Increased SMAD-phosphorylation at the carboxy-terminal region (SSXS) suggests a direct-effect of AA on SMAD-kinases ALK4/5/7 and ALK1/2/3/6. However, RTK-dependent phosphorylation of SMADs at the linker region by AA cannot be excluded [6], as the MEK/ERK-pathway has been implicated in AA-dependent increased collagen-production. Although AA has been shown to directly influence Nanog-upregulation in the presence of LIF [49,50] to promote ESC-stemness, it is unlikely that a similar mechanism is involved in AA-enhanced cardiomyogenesis, as the current study utilizes AA-treatments on differentiating EBs. Indeed, our data confirm that AA-augmented cardiomyogenesis is independent of AA’s antioxidant ability [5] and of its anti-proliferative role [13]. Our cell-cycle assessments, however, could not determine the effects of AA on subtypes of differentiated cells that would be expected in these heterogeneous cultures. We therefore cannot wholly exclude a possible angiostatic effect of AA [14,51,52] on specific differentiating-cell populations. Moreover, the data presented here following inhibition by dorsomorphin is not entirely consistent with published data [40]. While we cannot fully account for the discrepancy, one possible explanation may involve the experimental conditions, as the potential of mESCs to differentiate to CMs relies upon media-components (especially FBS) that are not always well defined. Additional variability may be attributable to the number of cells employed to form EBs and the timing of EB-plating [53,54].

Finally, to evaluate SMAD protein abundance (Day 3) and CM yields (Day 7+3) during differentiation, following AA-addition, we employed two inducible mESC-clonal lines with allowed conditional-expression of SMAD1- or SMAD2-proteins. As previously published, early (Day 2) and short-pulsed (6 to 24 hours) inductions of SMAD1 selectively expand the pre-hematopoietic mesoderm [42]; we performed conditional expression for 24 hours, from Day 2 to Day3 of differentiation. In these experiments, SMAD1 induction had a net positive effect on CM yield. Increased cardiomyogenesis was mirrored at the protein level (Day 3 of differentiation) by an increase in both phospho-SMAD1 and phospho-SMAD2, although only total SMAD1, but not total SMAD2 and SMAD3 proteins were up-regulated. This demonstrate the positive feedback of BMP-SMADs on TGFβ-SMADs in condition of BMP-pathway activation. In contrast, time-matched (Day 2) SMAD2 induced-expression showed no significant changes in SMAD-activation at Day 3, or CM production at Day 7+3 of differentiation, confirming the priority of BMP- over TGFβ-signals in mESC cardiomyogenesis. Perhaps most importantly, the inductive effect of AA on cardiomyogenesis was visible (at least in our experimental conditions) when SMAD1 was over-expressed, but not when SMAD2 was conditionally-manipulated. Instead, AA significantly decreased phospho-SMAD1/5/8 levels in condition of SMAD2 overexpression, consistent with a negative effect of AA on the TGFβ-parallel pathway.

**Fig 8:**
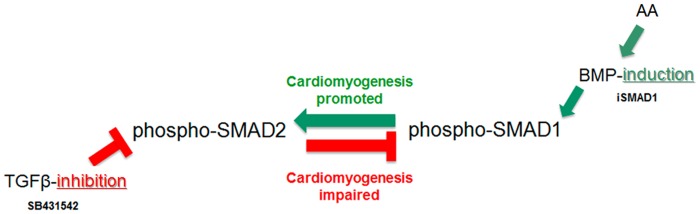
SMADs unidirectional cross-talk either promotes or impairs cardiomyogenesis. The inhibition of the TGFβ-signaling by SB431542 decreases the activation (phosphorylation) of the TGFβ-dependent SMAD2, as expected, but also downregulates the activation (phosphorylation) of the BMP-dependent SMAD1. SMAD2 negative feedback on SMAD1 impairs cardiomyogenesis. In contrast, the induction of the BMP-cascade by conditional expression of SMAD1, BMP2-stimulation or AA treatment, increases the activation (phosphorylation) of the BMP-dependent SMAD1, as expected, but also upregulates the activation (phosphorylation) of the TGFβ-dependent SMAD2. SMAD1 positive feedback on SMAD2 promotes cardiomyogenesis. These results show that SMAD1 is critical for cardiomyogenesis, and that AA acts in part through this positive unidirectional cross-talk.

In summary, AA-induced cardiomyogenesis is SMAD-modulation dependent. AA up-regulates phosphorylation of both TGFβ-SMAD2 and BMP-SMADs1/5/8 to increase cardiomyocyte yields from differentiating mESCs. At the protein level, the ratio of activated SMADs appears to be balanced by the existence of SMAD-crosstalk: an increase in phospho-SMAD1 (by BMP2 or SMAD1 conditional stimulation) has a positive feedback on phospho-SMAD2, while a reduction in phospho-SMAD2 (by SB431542) is mirrored by reduced phospho-SMAD1 levels (Fig 8). Moreover, AA acts synergistically with BMP2, but it antagonizes the TDGF1 positive effect on cardiomyogenesis by overstimulating the TGFβ-signaling. In addition, BMP-pathway activation is necessary and sufficient for cardiomyogenesis, as phospho-SMAD1-inhibition (dorsomorphin) and SMAD1-activation (BMP2 stimulation and SMAD1 overexpression in iSMAD1-EBs), either block or boost the cardiomyogenic program, respectively. From these results, we conclude that AA-enhanced cardiomyogenesis relies on AA’s ability to modulate the SMAD ratio by fine-tuning of the TGFβ-signaling pathways (canonical and lateral), possibly at the level of ALK5/ALK1.

## Supporting information

S1 Fig Generation and characterization of RFP6-mESC clonal line. **A. (i)** Schematic representation of the cloning strategy used to generate the RFP6 clone, a stable mESC-R1 derived cell line harboring the *ncx1*-driven dsRED transgene (RFP protein) and the Neomycin resistance (neoR) cassette under the *pgk* promoter; **(ii)** Flow cytometry assessment at Day 7+3 of the presence of RFP protein in RFP6-EBs when compared to its parental cell-line (R1-EBs). **B. (i)** Undifferentiated RFP6-ESC colonies selected with G418 (300 μg/ml); **(ii)** RFP6-cardiomyocytes (CMs) within embryoid bodies (EBs) at Day 7+9 showing presence of RFP protein; **(iii)** Immunofluorescent co-staining of α-Actinin (green) and RFP protein (red) in CMs at Day 7+4, 24 hours after FACsorting (Day 7+3 on RFP protein expression). Nuclei are labeled with Hoechst 33342. Bar 50 μm. **C. (i)** PCR analysis of pluripotency factors (*Pou5f1*, 81 bp and *Nanog* 81 bp) and the mesoderm marker *T* (71 bp) in RFP6-ESCs at Day 0; **(ii)** PCR assessment of the cardiac genes (*Nkx2*.*5* 181 bp; *Gata4* 113 bp; *α-MHC* 301 bp, *18S* 200 bp) in RFP6-CMs at Day 7+5 of differentiation, 48 hours after FACsorting (Day 7+3 on RFP protein expression).
(EPS)

S2 Fig Ascorbic acid-induction promotes cardiomyogenesis by increasing the expression of cardiac factors. Histogram of the relative protein abundance of TDGF1 (n = 3), GATA4 (n = 9) and T (n = 9) proteins in R1-EBs at Day 3 of differentiation, 24 hours after AA treatment (Day 2). *p<0.05 and **p<0.01 are relative to untreated control.
(EPS)

S3 Fig Induction of BMP- and TGFβ-pathways by conditional expression of SMAD1 and SMAD2, respectively, differentially affects cardiac protein expression. Western Blots of the cardiac markers GATA4 and T in SMAD-inducible (i) stem cell lines iSMAD1-ESCs and iSMAD2-ESCs, at Day 3 of differentiation (iSMAD-EBs). A. iSMAD1-EBs (n = 6) and B. iSMAD2-EBs (n = 3), were treated with doxycycline (Dox) for 24 hours from Day 2 to Day 3 of differentiation, to conditionally induce SMAD1 (A) or SMAD2 (B); AA treatment was performed at Day 2 of differentiation. *p<0.05, **p<0.01 and ***p<0.001 are relative to untreated iSMAD-mESC lines.
(EPS)
